# Effect of imbalance in dietary macronutrients on blood hemoglobin levels: a cross-sectional study in young underweight Japanese women

**DOI:** 10.3389/fnut.2023.1121717

**Published:** 2023-06-20

**Authors:** Yuko Tateishi, Reiko Ichikawa, Katsuya Suzuki, Yoshiro Kitahara, Yuki Someya, Yoshifumi Tamura

**Affiliations:** ^1^Institute of Food Sciences and Technologies, Ajinomoto Co., Inc., Tokyo, Japan; ^2^Sportology Center, Juntendo University Graduate School of Medicine, Tokyo, Japan; ^3^Department of Metabolism and Endocrinology, Juntendo University Graduate School of Medicine, Tokyo, Japan

**Keywords:** iron deficiency, nutrients, underweight women, hemoglobin, dietary habit

## Abstract

**Background:**

Iron deficiency and underweight are common nutritional problems among young Japanese women, many of whom show unhealthy dietary patterns owing to a desire for thinness. We conducted a cross-sectional analysis of the relationship between iron status, nutritional status, and dietary intake among young Japanese women with underweight to identify dietary risk factors for iron deficiency.

**Methods:**

Of the 159 young women (18–29 years of age) enrolled, 77 underweight and 37 normal-weight women were included in the study. They were further categorized into four groups based on quartiles of hemoglobin levels among all participants. Dietary nutrient intake was ascertained using a brief self-administered diet history questionnaire. Blood level of hemoglobin and nutritional biomarkers such as total protein, albumin, insulin-like growth factor-1 (IGF-1), and essential amino acids were measured.

**Results:**

In underweight, the multiple comparison test showed that dietary intakes of fat, saturated fatty acid, and monosaturated fatty acid were significantly higher and carbohydrate intake was significantly lower in the group with the lowest hemoglobin level, whereas intakes of iron were the same across groups. Multivariate regression coefficients suggested that replacing fat with protein or carbohydrates increased hemoglobin levels under isocaloric conditions. Additionally, significant positive correlations were observed between hemoglobin levels and nutritional biomarkers.

**Conclusion:**

Dietary iron intake did not change across different hemoglobin groups among Japanese underweight women. However, our results suggested that an imbalanced dietary macronutrient induces anabolic status and hemoglobin synthesis deterioration among them. Especially, a higher fat intake may be a risk factor for lower hemoglobin.

## 1. Introduction

Iron deficiency, which is one of the major causes of anemia, is globally one of the most common health and nutrition concerns. Women and children are prone to anemia, particularly in developing countries, because of poor nutritional status (1). The incidence of anemia is relatively high in young Japanese women, with the prevalence of anemia and iron deficiency without anemia reported to be 17% (2, 3) and 33–47% (4, 5), respectively. Iron deficiency causes dizziness, shortness of breath, headache, reduced performance, and cognitive dysfunction (6–10).

In Japan, a survey showed that 21% of the women in their 10s and 20s are underweight, while <10% of them are obese (11). The percentage of underweight women in Japan is prominently higher than those of other developed countries. Underweight can related to bone loss, low muscle mass, and iron deficiency. Being underweight has been identified as a risk factor for iron deficiency among women and children in Indonesia (12), India (13), and Bangladesh (14) because of poor nutritional status. Previous research evaluating the dietary habits of Japanese women showed that though the underweight women with a desire for thinness consume less cereal and rice, they eat more pastries, including candies and chocolates, than normal-weight women (15). This type of undesirable dietary habits can lead to health issues, including iron deficiency. Iron replacement is the standard treatment for iron deficiency anemia; however, high iron intake induces gastrointestinal side effects, and compliance and intolerance to oral iron preparations limit its efficacy. Hence, it is crucial to prevent or improve iron deficiency by adopting proper dietary behaviors.

There have been conflicting data in the nutritional studies among Japanese with iron deficiency. A study (16) showed that the dietary iron intake is lower in iron deficiency than normal; however, other studies (17, 18) found that dietary iron intake did not change between the two groups. Thus, other factors including nutrients other than iron may be involved in the prevalence of iron deficiency in Japanese women. Some nutritional studies have indicated that dietary intakes of energy, protein (19, 20), and vitamin A (21, 22) are related to the prevalence of iron deficiency. In addition, some nutrients have been shown to reinforce iron absorption and its biological functions. For example, vitamin B12 and folic acid are required for erythropoiesis (23); vitamin C increases non-heme iron absorption and modulates the transferrin-iron uptake pathway (24); and sufficient intake of energy and protein are necessary for effective erythropoiesis (25, 26).

To identify dietary risk factors for iron deficiency other than dietary iron, we conducted an exploratory analysis of cross-sectional study for investigating the relationship between blood iron status, dietary nutrients, and nutritional status in young Japanese underweight women aged 18–29 years.

## 2. Materials and methods

### 2.1. Participants

A total of 159 women, healthy, young, and aged 18–29 years were enrolled, of which 101 were underweight with body mass index (BMI) between 15.4 and 18.5 kg/m^2^, and 58 were normal-weight with BMI between 18.5 and 26.0 kg/m^2^ through two outsourcing companies (Clinical Trial, Tokyo, Japan, and SOUKEN, Tokyo, Japan), from November 2018 to December 2019, as previously reported in our study (27). BMI criteria was based on the definition of World Health Organization (WHO). Although in the previous study, 3 participants with BMI of <16 kg/m^2^, and 2 participants with BMI ≥23 kg/m^2^ were excluded, we included them in this study. Among underweight women, we excluded 3 and 21 participants because they took iron supplements and had irregular menstruation or amenorrhea, respectively. Among the normal-weight women, 4 and 17 participants were excluded due to iron supplementation and irregular menstruation, respectively. Finally, 77 underweight and 37 normal-weight women were included (Supplementary Figure S1). The study was conducted in accordance with the Declaration of Helsinki, and the protocol was approved by the Institutional Review Boards of Juntendo University (No. 2018139) and Ajinomoto Co., Inc. (No. 2018–022).

### 2.2. Dietary assessment

Nutrient dietary intake was determined using a brief self-administered diet history questionnaire (BDHQ) (28, 29). The BDHQ is a four-page fixed-portion questionnaire estimating the dietary intake of 46 food and beverage items in the previous month. The dietary intake for energy and selected nutrients, including protein, fat, fatty acids, iron, zinc, vitamin C, vitamin A, vitamin 12, and folic acid were estimated using an *ad hoc* computer algorithm for the BDHQ. Nutrient intake of participants was calculated from November 2018 to December 2019.

### 2.3. Blood analysis

Blood samples were collected from all participants after overnight fasting, and the levels of hemoglobin (Hb) and hematocrit (Ht) were measured. Plasma and serum samples were collected for the analysis of serum iron, total iron binding capacity (TIBC), ferritin, total protein (TP), albumin (ALB), and insulin growth factor (IGF-1). Measurement of these parameters were performed by SRL, Inc. (Tokyo, Japan). Plasma amino acids were measured using high-performance liquid chromatography-electrospray ionization-mass spectrometry by precolumn derivatization with Agilent 1,100 series (Agilent Technologies, Waldbrunn, Germany), as described in Shimbo et al. (30). All measurements were performed from November 2018 to December 2019.

### 2.4. Statistical analysis

Data on age, height, weight, BMI, Hb, Ht, serum iron, and TIBC were expressed as mean ± SD, and ferritin was expressed as median ± interquartile range (IQR). Mann–Whitney U test was applied to compare the values between underweight and normal-weight. The participants were categorized into four groups based on the quartiles of Hb levels among all participants, including underweight and normal-weight women (Q1, Q2, Q3, and Q4, respectively). The Hb levels of four group were 8.9–12.3, 12.4–12.8, 12.0–13.4, and 13.5–14.4 g/dL among underweight, and 10.3–12.3, 12.4–12.8, 12.9–13.3, and 13.5–15.0 g/dL among normal-weight. Dietary nutrients intake, TP, ALB, IGF-1, and essential amino acids (EAA) in each group are expressed as median ± IQR. Energy-adjusted data with a residual method were used as dietary nutrients intake. The percentage of energy from each macronutrient was calculated as carbohydrate, protein, and fat containing 4, 4, and 9 kcal per gram, respectively. Mann–Whitney U test with Holm correction was conducted to compare the dietary nutrients intake across four groups. Pearson’s correlation coefficients, *t*-values, and *p*-values were determined to investigate the linearity relationships between blood levels of hemoglobin and nutritional biomarkers.

Substitution analysis (31) was conducted to evaluate the association between intake of macronutrients and Hb levels. Substitution analysis emulates feeding studies which are used to identify the differences in the intake of the specified macronutrients that contribute to energy intake which may be accompanied by variations in the intake of other macronutrients. For example, the effect on Hb level by substituting fat for protein while holding the total consumption of fat, protein, and carbohydrate can be estimated either by leaving only protein out of the multivariable model including consumption of fat, carbohydrate, and the total energy intake (“leave-one-out” model). The coefficients from these models can be interpreted as the estimated effect of replacing a specific percentage of protein with the same percentage of fat. The Hb levels was applied in the models as outcome variable. In addition, the intake of each macronutrient with energy adjustment (g/day) and energy intake (kcal/day) was applied as explanatory variables in the crude models, and the models were further adjusted for age and BMI, which are considered as influence factors on hemoglobin levels. In addition, the ridge regression method, which is used in other nutritional studies (32, 33) to reduce multicollinearity, was applied in the models. The R package *ridge* was used for this, and the ridge parameter was selected automatically using the proposed method (34).

For supplementary tables, Fisher’s exact test was used to compare the prevalence of anemia between the groups. Dietary nutrients intake (not energy adjusted) between two groups of underweight and normal-weight were compared by Mann–Whitney U test. Mann–Whitney U test with Holm correction was conducted to compare the blood levels of nutritional biomarkers (TP, ALB, IGF-1 and EAA) across four groups. All statistical analyses were performed using R version 4.0.2, and statistical significance was set at *p* < 0.05.

## 3. Results

Participants’ characteristics are presented in Table 1. The weight, BMI, and Hb of underweight women were significantly lower than those of normal-weight women. Blood Ht, ferritin, serum iron, and TIBC were similar between the two groups. World Health Organization defines anemia in non-pregnant women as Hb <12.0 g/dL; as shown in Supplementary Table S1, anemia prevalence was not changed between two groups.

**Table 1:** General characteristics of the participants by BMI categories.

Variable	Underweight (*n* = 77)	Normal weight (*n* = 37)	*p-*value
Age (years)	24.0 (3.0)	22.6 (3.3)	0.021
Height (cm)	159.6 (5.2)	158.6 (6.2)	0.483
Weight (kg)	44.1 (3.1)	52.7 (5.9)	<0.001
BMI (kg/m^2^)	17.3 (0.7)	20.9 (1.6)	<0.001
Hb (g/dL)	12.8 (0.9)	13.1 (1.0)	0.021
Ht (%)	39.3 (2.7)	40.3 (2.7)	0.081
Ferritin (ng/mL)	19.9 (18.5)	24.0 (12.9)	0.146
Serum iron (μg/dL)	81.6 (31.1)	88.4 (32.3)	0.227
TIBC (μg/dL)	332.7 (43.7)	336.9 (33.9)	0.511

Values are expressed as median (IQR) for ferritin and mean (SD) for other variables. Mann–Whitney U test was used to calculate the *p*-values. BMI, body mass index; Hb, hemoglobin; Ht, hematocrit; TIBC, total iron-binding capacity.

After categorized by the quartiles of Hb levels which is the key indicator for iron status, nutritional dietary intake among normal-weight women did not differ across the four groups (data not shown); hence, the prevalence of iron deficiency did not depend on dietary factors in normal-weight. On the other hands, as presented in Table 2, intake of fat (g/day), percentage energy from fat (%Energy), saturated fatty acid (g/day), and monounsaturated fatty acids (g/day) were significantly higher in Q1 than Q2 (*p* = 0.006, *p* = 0.001, *p* = 0.021 and *p* = 0.007, respectively) among underweight women. Conversely, intake of carbohydrate (g/day) and percentage energy from carbohydrate (%Energy) was significantly lower in Q1 than Q2 (*p* = 0.016 and *p* = 0.002, respectively). From the dietary assessment by the BDHQ, we found that lower carbohydrate intake derived from lower intakes of cereal, and higher intake of fat was mainly due to higher intake of fats/oils, meats, and dairy products. Dietary intake of iron, vitamins and folic acid was not different across the four groups in underweight women.

**Table 2:** Comparison of the intake of calories and nutrients between the four groups of underweight women.

Nutrients	Unit	Q1 (8.9–12.3) (*n* = 24)	Q2 (12.4–12.8) (*n* = 19)	Q3 (12.9–13.4) (*n* = 19)	Q4 (13.5–14.4) (*n* = 15)
Energy	(kcal/day)	1244.2 (355.0)	1389.3 (593.9)	1332.7 (493.5)	1356.0 (295.2)
Protein	(g/day)	50.7 (9.9)	47.5 (9.1)	48.9 (9.8)	43.9 (10.4)
Fat	(g/day)	46.9 (8.5)^a^	40.7 (7.0)^b^	44.7 (10.8)^ab^	39.8 (14.2)^ab^
SFA	(g/day)	12.8 (4.4)^a^	10.7 (2.1)^b^	11.6 (3.2)^ab^	10.6 (5.1)^ab^
MUFA	(g/day)	17.1 (4.1)^a^	14.8 (2.5)^b^	16.6 (3.9)^ab^	15.3 (4.6)^ab^
PUFA	(g/day)	10.3 (2.6)	9.4 (1.6)	10.2 (2.3)	10.5 (2.7)
Carbohydrate	(g/day)	173.5 (31.1)^a^	190.4 (17.4)^b^	181.3 (24.4)^ab^	179.4 (35.4)^ab^
Iron	(mg/day)	5.5 (1.7)	5.3 (0.9)	5.0 (1.1)	5.0 (1.6)
Zinc	(mg/day)	6.1 (0.7)	5.9 (0.8)	5.7 (0.9)	5.3 (1.1)
Vitamin C	(mg/day)	74.6 (44.7)	66.7 (38.3)	60.0 (40.3)	59.9 (38.9)
Vitamin A	(μgRAE/day)	389.8 (144.1)	525.5 (432.1)	433.6 (217.7)	354.6 (174.8)
Vitamin B12	(μg/day)	5.9 (3.3)	5.3 (1.7)	5.2 (3.0)	4.9 (2.4)
Folic acid	(μg/day)	200.7 (100.3)	217.5 (44.8)	188.9 (117.2)	185.0 (75.9)
Macronutrient intake (%Energy)					
Protein	%	15.3 (3.6)	14.0 (2.8)	14.4 (2.9)	12.8 (2.8)
Fat	%	32.2 (5.5) ^a^	26.9 (4.3) ^b^	29.5 (7.4)^ab^	26.5 (9.1)^ab^
Carbohydrate	%	48.8 (5.6) ^a^	56.2 (4.4) ^b^	53.8 (10.1)^ab^	53.0 (13.2)^ab^

Values are expressed as medians (IQR). Energy adjustment was performed by using the residual method for each participant. The same alphabet show no significant difference, while the groups with different alphabets show significant difference by Mann–Whitney U test with holm correction. SFA, saturated fatty acid; MUFA, monosaturated fatty acid; PUFA, polyunsaturated fatty acid; RAE, retinol activity equivalents.

To examine the effect of dietary macronutrient proportion on Hb level in the isocaloric context, we constructed a multivariate model including intake of two macronutrients and energy intake as explanatory variables, excluding one macronutrient. As presented in Table 3, the coefficients for fat were significantly negative in both models with protein excluded (*p*-value for crude and adjusted models; *p* = 0.025 and *p* = 0.045), and with carbohydrate excluded (*p*-value for crude and adjusted models; *p* = 0.012 and *p* = 0.016). In contrast, no significant effect was observed on Hb levels in the model with the fat excluded.

**Table 3:** Linear regression analysis of macronutrient intake with respect to Hb level of underweight women (*n* = 77).

		Crude model		Adjusted model	
	Macronutrients in the model	Standardized coefficient (SE)	*p*-value	Standardized coefficient (SE)	*p*-value
Model including fat and carbohydrate	Fat	−1.55 (0.69)	0.025*	−1.40 (0.70)	0.045*
	Carbohydrate	0.38 (0.69)	0.582	0.38 (0.70)	0.589
	Energy	−0.28 (0.69)	0.682	−0.31 (0.69)	0.655
Model including fat and protein	Fat	−1.71 (0.68)	0.012*	−1.55 (0.64)	0.016*
	Protein	−0.05 (0.68)	0.936	0.08 (0.64)	0.906
	Energy	−0.27 (0.67)	0.682	−0.28 (0.64)	0.660
Model including protein and carbohydrate	Protein	−0.25 (0.57)	0.663	−0.04 (0.64)	0.946
	Carbohydrate	1.01 (0.57)	0.080	1.12 (0.64)	0.083
	Energy	−0.24 (0.59)	0.688	−0.29 (0.64)	0.657

The crude models were adjusted for energy intake (kcal). The models were further adjusted for BMI and age. Hb, hemoglobin. *p < 0.05.

Supplementary Table S2 shows difference in levels of nutritional biomarkers between the four groups of underweight women, whereas, Table 4 shows the association between blood levels of nutritional biomarkers and Hb levels. As shown in Supplementary Table S2, blood levels of TP and ALB were significantly lower in Q1 and Q2 than Q4. Additionally, the levels of IGF-1 were significantly lower in Q1 than Q4. Table 4 shows that all nutritional biomarkers were positively correlated with Hb levels with weak to mild correlation coefficients with *r* = 0.26 to 0.47 (*p* < 0.001, *p* < 0.001, *p* < 0.001, and *p* = 0.024 for TP, ALB, IGF-1, and EAA respectively).

**Table 4:** Correlation between nutritional biomarker levels and hemoglobin levels among underweight women (*n* = 77).

	*r*	*t*	*p*-value
TP (g/dL)	0.46	4.57	<0.001
Albumin (mg/dL)	0.45	4.42	<0.001
IGF-1 (ng/mL)	0.42	4.04	<0.001
EAA (μmol/L)	0.26	2.30	0.024

Pearson’s correlation coefficient, *t*-value, and *p*-value were determined among all underweight participants. TP, total protein; IGF-1, insulin-like growth factor-1; EAA, essential amino acids.

## 4. Discussion

We found that increased fat intake may be a dietary risk factor for low Hb levels, particularly in underweight women. This is the first study investigating the relationship between macronutrient intakes and Hb levels in young Japanese women. In our study, the energy intake of underweight women was 65% of dietary reference intake (DRI) in Japan (2,000 kcal/day). However, the fat intake in underweight women was as high as that in normal-weight women (Supplementary Table S3). Interestingly, median intake of fat was highest in the group with the lowest Hb level (Q1) across the four groups, and was also higher than DRI (20–30% Energy) in underweight women. Our BDHQ data suggested that higher fat intake was mainly due to a higher consumption of fats/oils, meats and dairy products. In addition, median intake of carbohydrate in Q1 was the lowest across the four groups, mainly due to lower cereal consumption. From these results, it was observed that participants in the Q1 group of underweight, which has the highest risk of iron deficiency anemia, had the most unhealthy dietary habits. Among Q2, Q3, and Q4, the groups showed normal hemoglobin levels of >12.4 g/dL, and the dietary nutrients intake were not significantly different. Multivariate analysis showed that increasing the fat proportion adversely affected the Hb levels. This result implies that Hb levels may not improve even when energy intake is supplemented with fat. Although further analyses are needed to understand the causal relationship between fat intake and iron status, some studies have shown a significant association between them. A high-fat diet was shown to suppress iron absorption (35) and lead to abnormalities in iron metabolism in rodents (36). Furthermore, increasing the dietary percentage energy from carbohydrate to fat was shown to improve dietary protein usage (37), and this may influence Hb synthesis because amino acids are required for intra-erythroblast protein synthesis (38). As for the intake of iron and vitamins, which is known to affect iron bioavailability, their intake levels were the same between the different Hb groups.

We found that blood levels of Hb and nutritional biomarkers (TP, albumin, IGF-1, and EAA) were positively correlated. Interestingly, among these biomarkers, IGF-1 which is a growth hormone with anabolic functions was significantly lower in underweight than in normal-weight (median values of 156.0 ± 48.0 ng/mL and 191.1 ± 48.3 ng/mL for underweight and normal-weight, respectively; *p* < 0.001 by the Mann–Whitney U test). This result suggested that the anabolic functions in underweight women are in declining trend. In developing countries, where energy and protein intake restriction is commonly observed, deficiency of dietary protein may induce low levels of serum IGF-1 (39). Similarly in developed countries, dietary protein level has been reported to be positively correlated with serum IGF-1 level (40, 41). However, in our study, dietary protein level was not reflected in serum IGF-1 levels among Japanese underweight women; thus, the anabolic function of dietary protein may be reduced among them. In young Japanese women who desire to be thin, their calorie intake is restricted similar to that commonly observed in developing countries, but their dietary pattern is characteristic of lowered carbohydrate intake and higher fat intake. This imbalanced diet particularly observed in Q1 group may be one of the factors responsible for reduced anabolic status, and further suppression of Hb synthesis. On the other hand, unlike nutritional biomarkers, dietary intake of fat or carbohydrate did not change between the groups of Q2, Q3, and Q4. There must be other factors besides dietary nutrient involved in anabolic status and Hb synthesis, and further research is needed to elucidate the mechanisms of it.

Our study demonstrated the potential association between dietary macronutrient balance, anabolic status, and Hb level (Supplementary Figure S2). However, this study had certain limitations. First, analysis should focus on iron deficiency anemia and iron deficiency without anemia (defined as low ferritin levels), but we could not carry out this because of limited number of participants. This study was the exploratory cross-sectional study, and proper sample size for analysis was not considered. Further studies with larger cohort with proper sample size are needed for verifying our results and analyzing the relationship between serum ferritin and dietary nutrient intake. Second, dietary data were obtained using BDHQ. As actual dietary habits were unobserved, the results should be interpreted with caution, although the validity of the BDHQ appears reasonable. Third, we could not identify the food that varied significantly between the different Hb groups. Further analysis of food intake is required to propose appropriate dietary habits to prevent iron deficiency. Forth, iron deficiency is influenced not only by nutritional status, but also by inflammation or exercise status. To understand the proper dietary behaviors to prevent iron deficiency according to individual situation, it is necessary to analyze including these risk factors.

In conclusion, dietary levels of iron did not change across different Hb groups among Japanese underweight women. “On the other hand, an increased proportion of energy intake from fat may be one of the risk factors for lower anabolic status. Furthermore, our study suggested that improving anabolic status may be crucial for maintaining high Hb levels”. Further studies are necessary to explore in more detail the dietary factors and foods that control iron deficiency, especially in young Japanese women.

## Data availability statement

The original contributions presented in the study are included in the article/Supplementary material, further inquiries can be directed to the corresponding authors.

## Ethics statement

The studies involving human participants were reviewed and approved by the Institutional Review Board and Ethics Committee of Juntendo University and Ajinomoto Co., Inc. The patients/participants provided their written informed consent to participate in this study.

## Author contributions

YoT, YS, and YK designed the study, collected, and analyzed. YuT and RI performed the statistical analysis. YuT prepared the manuscript. KS, YK, YS, and YoT helped to draft the manuscript with its critical review. All authors gave final approval on the manuscript.

## Funding

This study received funding from Ajinomoto Co., Inc.

## Conflict of interest

YuT, RI, KS, and YK were employed by Ajinomoto Co., Inc.

The remaining authors declare that the research was conducted in the absence of any commercial or financial relationships that could be construed as a potential conflict of interest.

## Publisher’s note

All claims expressed in this article are solely those of the authors and do not necessarily represent those of their affiliated organizations, or those of the publisher, the editors and the reviewers. Any product that may be evaluated in this article, or claim that may be made by its manufacturer, is not guaranteed or endorsed by the publisher.

## Supplementary material

The Supplementary material for this article can be found online at: https://www.frontiersin.org/articles/10.3389/fnut.2023.1121717/full#supplementary-material
